# The biocontrol nematode *Phasmarhabditis hermaphrodita* infects and increases mortality of *Monadenia fidelis*, a non-target terrestrial gastropod species endemic to the Pacific Northwest of North America, in laboratory conditions

**DOI:** 10.1371/journal.pone.0298165

**Published:** 2024-03-21

**Authors:** Dee Denver, Dana K. Howe, Andrew J. Colton, Casey H. Richart, Rory J. Mc Donnell

**Affiliations:** 1 Department of Integrative Biology, Oregon State University, Corvallis, OR, United States of America; 2 Department of Crop and Soil Science, Oregon State University, Corvallis, OR, United States of America; CPRI: Central Potato Research Institute, INDIA

## Abstract

Inundative biological control (biocontrol) efforts in pest management lead to the mass distribution of commercialized biocontrol agents. Many ‘biocontrol gone awry’ incidents have resulted in disastrous biodiversity impacts, leading to increased scrutiny of biocontrol efforts. The nematode *Phasmarhabditis hermaphrodita* is sold as a biocontrol agent on three continents and targets pest gastropods such as *Deroceras reticulatum*, the Grey Field Slug; *P*. *hermaphrodita* is not presently approved for use in the United States. Investigations into the potential for *P*. *hermaphrodita* to infect non-target gastropod species of conservation relevance, however, are limited. We examined the effects of three strains of *P*. *hermaphrodita* on mortality in *Monadenia fidelis*, the Pacific Sideband, a snail species endemic to the Pacific Northwest of North America, in laboratory conditions. Across a 71-day laboratory infectivity assay, snails exposed to each of the three nematode strains, each analyzed at two doses, experienced a mean 50% mortality by days 20–42. All nematode-treated snails were dead by the end of the study. By contrast, 30/30 water-control snails experienced no mortality. Nematodes killed smaller, juvenile-stage snails significantly faster than those in larger and more developmentally advanced hosts. Our results provide direct evidence that the biocontrol nematode *P*. *hermaphrodita* infects and kills *M*. *fidelis*, a non-target gastropod species endemic to the Pacific Northwest, in laboratory conditions. This study suggests that introduction of *P*. *hermaphrodita* to new ecosystems might negatively impact endemic gastropod biodiversity and advocates for further investigation of non-target effects, including in conditions closer to the natural environments of non-target species.

## Introduction

Human activities pose diverse threats to biodiversity across the planet. Some taxonomic groups are more susceptible to extinction than others. For example, terrestrial gastropods (slugs and snails) make up more than one-third of the total documented animal species extinctions since the year 1500 [1]. In addition to factors such as rising global temperatures and habitat loss that impact all biodiversity, gastropod species have historically suffered from poorly designed and executed biological control (biocontrol) efforts. For example, the intentional introduction of the snails *Euglandina* spp. and the flatworm *Platydemus manokwari* for biocontrol purposes is implicated in the extinction of hundreds of snail species endemic to Hawai’i and other Pacific islands [2]. Despite efforts to better design and execute biocontrol programs that are less harmful to the ecosystems in which they are deployed, including inundative strategies that avoid some of the pitfalls of prior classical biocontrol approaches, unintended impacts on endemic non-target species continue to be understudied and difficult to predict [3–5].

The genus *Phasmarhabditis* includes numerous nematode species that parasitize a variety of gastropods; one unnamed species in this nematode genus infects and kills earthworms [6–11]. A recent molecular-phylogenetic and taxonomic analysis suggests that nematodes previously and currently described under the junior synonym *Phasmarhabditis* should be renamed as *Pellioditis* [11]. For this article, we decided to continue using *Phasmarhabditis* given the extensive use of this name in past biocontrol and biological research literature. *Phasmarhabditis hermaphrodita* is a gastropod-parasitic nematode species that is lethal to many terrestrial slugs and snails, and has been developed as a commercially available biocontrol agent in Europe, Canada, and Kenya. To carry out infection, nematode infective juveniles (third larval dauer stage) enter host gastropods via the dorsal integumental pouch beneath the mantle and subsequently reproduce in large numbers (250–300 eggs produced per nematode) inside the host [6]. Smaller and less developmentally advanced gastropod hosts are observed to be more susceptible to nematode infection as compared to larger adults of the same species [12–14]. Some snail species are observed to trap nematodes with their shells as a defense mechanism [15]. Recently, *Phasmarhabditis californica* replaced *P*. *hermaphrodita* in the NemaSlug® 2.0 product that is currently sold in England, Scotland, and Wales. In the original NemaSlug® 1.0 product, *P*. *hermaphrodita* was formulated with a bacterial associate, *Moraxella osloensis*, that has been thought to play a role in host killing, though recent evidence has challenged this idea [16]. Although biocontrol products containing *P*. *hermaphrodita* or related nematodes are not currently permitted for sale in the United States, there is growing interest in this system due to the increasing impacts of pest gastropods, such as the Grey Field Slug *Deroceras reticulatum*, on agricultural systems [17, 18]. Though not permitted for sale in the U.S., *P*. *hermaphrodita* has been discovered and isolated from a variety of slug species in California and Oregon over the last decade [9].

Knowledge on the effects of *P*. *hermaphrodita* and related biocontrol nematodes on non-target gastropod species is limited, especially outside of Europe. An early study focused on *P*. *hermaphrodita* non-target effects analyzed seven European snail species and found 2/7 to experience significant mortality when exposed to the biocontrol nematodes, as compared to untreated controls [19]. Further research has shown that some gastropod species appear to not be susceptible to *P*. *hermaphrodita*, but that there are other taxonomically diverse non-target species that suffer significant mortality when exposed to these biocontrol nematodes [20–22]. A recent review article [15] listed 12 different target snail species as not susceptible to *P*. *hermaphrodita* and 12 target snail species as susceptible. Grewal et al. 2003 found that *P*. *hermaphrodita* infections (using the NemaSlug® 1.0 strain) caused significant mortality in two North America-native slug species, as compared to controls [23]. Despite this mixed picture and the continuing paucity of knowledge on the host range of *P*. *hermaphrodita*, especially in parts of the world outside Europe where non-target testing is very limited, *P*. *hermaphrodita* is often characterized as safe for non-target organisms and the environment [2].

The Pacific Northwest region of the United States is considered a gastropod biodiversity hotspot, with many endemic and taxonomically diverse species occupying its temperate, high-precipitation ecosystems [24]. The Pacific Sideband (*Monadenia fidelis*) is one such Pacific Northwest endemic snail, with a range that extends from southwest British Columbia, Canada, to northern California. Within this range, *M*. *fidelis* primarily occurs west of the Cascade Mountains, where they are associated with relatively undisturbed deciduous and mixed forest areas but occur in a wide variety of habitats; they are not typically found in managed agro-ecosystems [25–27]. The genus *Monadenia* (Xanthonychidae) contains 19 recognized species, all of which are endemic to western North America. There are eight recognized subspecies of *M*. *fidelis* [28], though a recent population-genomic analysis of *Monadenia* spp. in the Pacific Northwest revealed extensive admixture and gene flow between species and subspecies [29]. Although *M*. *fidelis* is widespread throughout its natural range, one subspecies, *Monadenia fidelis minor*, was petitioned for federal listing under the Endangered Species Act [29].

In this study, we aimed to shed initial light on the potential impacts of *P*. *hermaphrodita* on Pacific Northwest-endemic terrestrial gastropods. We investigated the susceptibility of *M*. *fidelis* to three different strains of *P*. *hermaphrodita*, each originally isolated in Oregon [9], through lab infectivity assays. We also examined snail mortality as a function of shell size, serving as a proxy for stage of development. Our findings provide important early insights into the potential impacts of commercialized biocontrol nematodes on endemic gastropod biodiversity in the United States, and suggest that consideration of host size and/or stage of development is essential for accurate and robust assessments of gastropod susceptibilities to biocontrol nematodes in lab infectivity trials.

## Materials and methods

### Nematodes and snails

**Fig 1:**
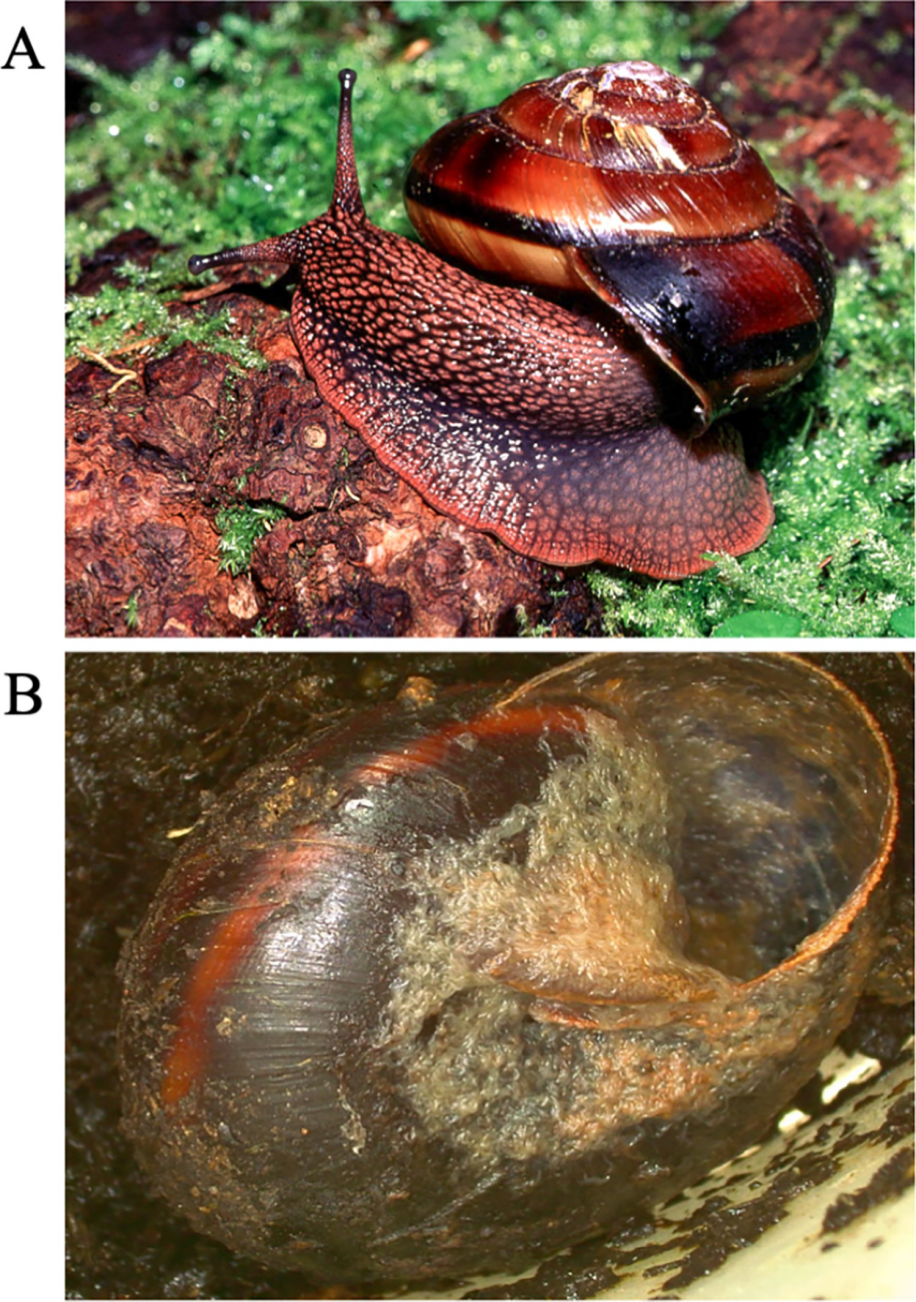
*M*. *fidelis* living in its natural ecology and after nematode infection. (A). Living specimen of *M*. *fidelis* in its natural ecology. Reprinted from CalPhotos under a CC BY license, with permission from William P. Leonard, original copyright 1999. The image shown is similar but not identical to the original image and is therefore for illustrative purposes only. (B). *P*. *hermaphrodita*-infested specimen from a laboratory infectivity trial. Thousands of *P*. *hermaphrodita* (strain DL 309) nematodes are visible in the center of the image.

We collected samples of *M*. *fidelis* between May 20–22, 2019 at Bald hill Natural Area, Corvallis OR, USA (44.5683343, -123.3316344). Permits were not required for the collection of *M*. *fidelis* in Oregon, as per Oregon Department of Agriculture policies regarding terrestrial gastropods. All specimens were kept in the laboratory in plastic containers at 18°C and fed organic carrot as described in Mc Donnell et al. 2020 for 3–4 days before the infection trial began [30]. The diameter of each specimen’s shell was measured using a Vernier digital caliper. An image of *M*. *fidelis* in its natural ecology is shown in Fig 1A.

Three Oregon-derived strains of *P*. *hermaphrodita*, DL300, DL307 and DL309, were used in this study. Each strain, grown in large numbers in the laboratory on 30 to 40 standard NMG plates with xenic co-culturing bacteria as food source, was collected, washed and counted following the procedures outlined in Mc Donnell et al. 2020 and applied again in Mc Donnell et al. 2021 [30, 31]. Mixed stage nematodes of each strain were aliquoted into individual 5 mL suspensions for high-dose (H) treatments (~40,000 nematodes) and low-dose (L) treatments (~20,000 nematodes) for the infectivity trials.

### Infectivity assays

Infectivity trials occurred in 12 cm diameter lidded plastic containers with 25 g of autoclaved soil as described in Mc Donnell et al. 2020 [30]. Into each container, one strain of nematode was added in 5 mL of water, either 20,000 nematodes for a low rate or 40,000 nematodes for a high rate. Three snails were added to each container, one with a small shell and two with larger shells. Snails were fed a slice of organic carrot every eight days; carrot slices were removed after 24 hours. A total of five replicates per strain per rate and 10 replicates of nematode free controls were used. All the infection trial containers were maintained in a growth chamber at 18°C with a 12-hour photoperiod. Each container was checked for mortality daily for the first 21 days, then weekly until day 71 post-inoculation. Snail survival assessment followed the protocols outlined in Mc Donnell et al. 2020 [30].

### Statistical analyses

For the snail survival data, Lethal Time 50 (LT50) and fiducial limits (95%) were calculated using probit analysis. It was not necessary to use Abbott’s formula to correct the treatment data as control mortality was 0%. Statistically significant differences (P<0.05) were considered as when there was no overlap between the 95% fiducial limits. The relationships between shell diameter and the number of days survived by individual snails for each nematode strain/rate were evaluated using Spearman rank correlations. IBM® SPSS® Version 24 was used for all analyses.

## Results

### Effects of *P*. *hermaphrodita* on *M*. *fidelis* mortality

**Fig 2:**
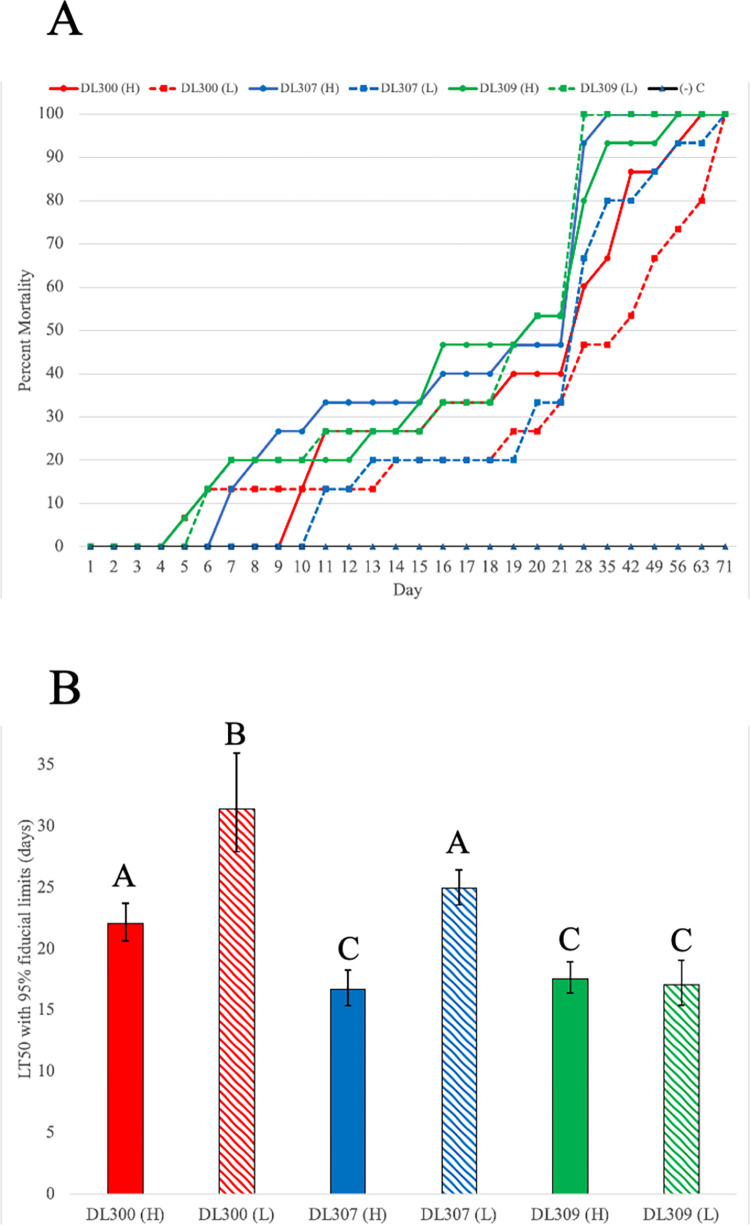
Impacts of *P*. *hermaphrodita* on *M*. *fidelis* mortality. Percent mortality observed (y-axis) as a function of number of exposure days (x-axis) is shown in (A). Results are shown for three *P*. *hermaphrodita* strains (DL300, red; DL307, blue; DL309, green), and for high (solid lines) and low (dashed lines) exposure rates. LT50 values for the six different strain x rate treatments are shown in (B). Error bars indicate 95% fiducial limits; A, B, and C above bars indicate three different confidence groupings based on overlapping fiducial limits.

All three Oregon strains of *P*. *hermaphrodita* (DL300, DL307 and DL309) were found to be lethal to the Pacific Sideband snail, *M*. *fidelis*, in the laboratory infectivity assays (Figs 1B and 2A, S1 File). The first snail mortalities occurred on the fifth day post-inoculation, in the DL300 (L) treatment and the DL309 (H) treatment. The DL309 (L) treatment was the first to show complete snail mortality, at day 28 post-inoculation.

**Fig 3:**
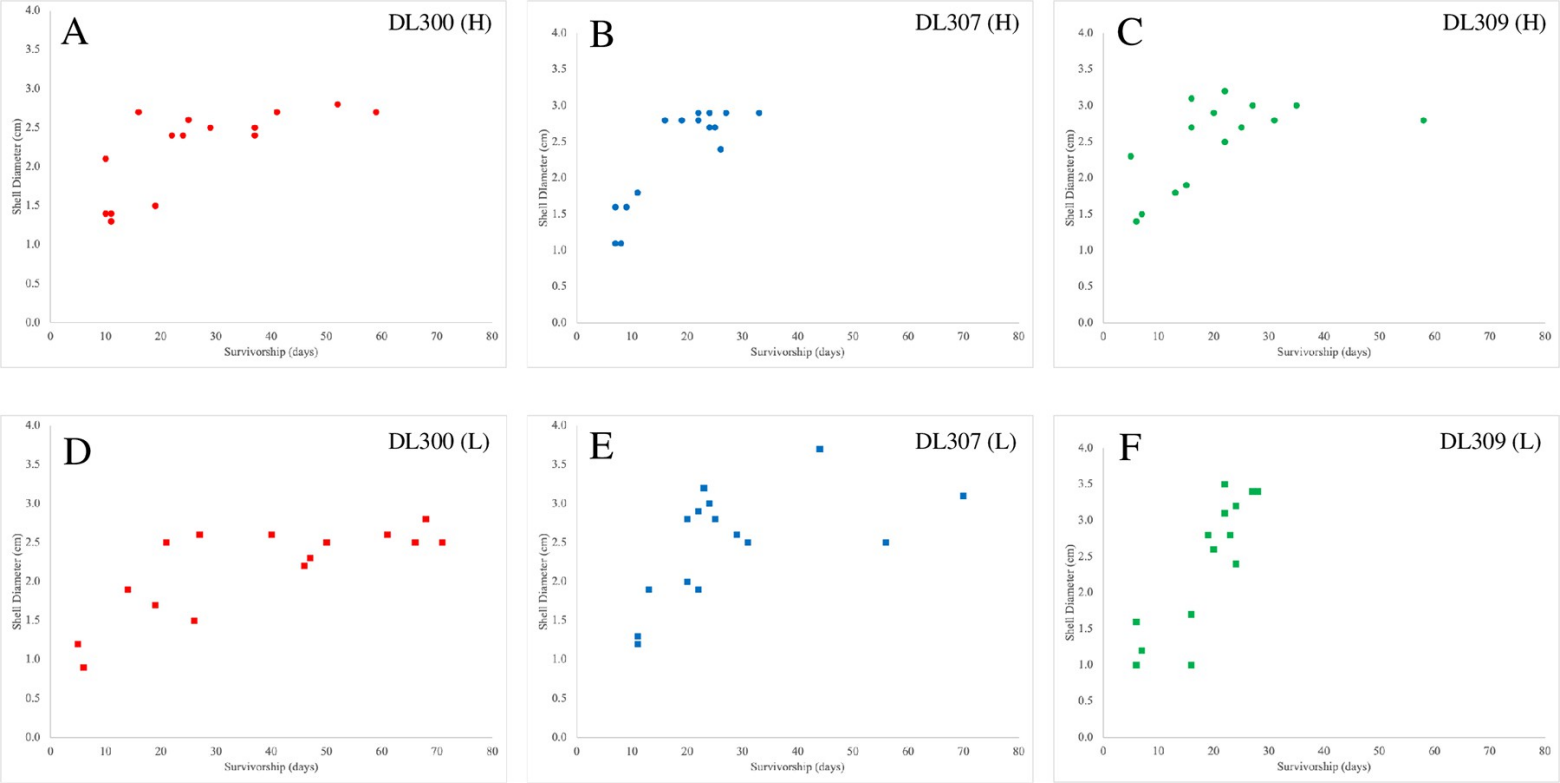
Impacts of *P*. *hermaphrodita* on *M*. *fidelis* survivorship as a function of snail shell diameter. Results for high-rate treatments are shown on top (A-C) and results for low-rate treatments are shown on the bottom (D-F). Statistically significant Spearman correlations were observed for all six [strain x dose-rate] treatments: DL300 (H) r = 0.778, p = 0.001; DL 300 (L) r = 0.709, p = 0.005; DL 307 (H) r = 0.730, p = 0.002; DL 307 (H) r = 0.634, p = 0.011; DL 309 (H) r = 0.667; p = 0.007; DL 309 (L) r = 0.801; p < 0.001.

Snails survived for significantly fewer days in all the nematode treatments compared to the water controls, where there was 100% snail survival through the course of the assay (Fig 2A). LT50 was reached between days 20–42 in the six experimental treatments. In the DL309 (H) and (L) treatments, LT50 was reached at the same time, day 20 post-inoculation. DL300 (H) reached LT50 ~14 days before DL300 (L); and DL307 (H) reached LT 50 ~7 days before DL307 (L). Based on non-overlapping 95% fiducial limits, three different significance groupings were observed among the six [strain x dose-rate] experimental treatments (Fig 2B).

### Snail mortality and shell diameter

We observed a size-dependent relationship between snail shell size and the time it took *P*. *hermaphrodita* to cause mortality. Infected snails with smaller diameter shells died more quickly than snails with larger diameter shells. This correlation was statistically significant for all three *P*. *hermaphrodita* strains as estimated by Spearman rank correlations, for both the high- and low-dose treatments (Fig 3).

## Discussion

The experiments described here demonstrate that *P*. *hermaphrodita*, a commercialized biocontrol species targeting pest slug species such as *D*. *reticulatum*, is lethal to *M*. *fidelis*, a non-target snail species endemic to the Pacific Northwest of North America, in laboratory conditions. Snail lethality was consistently observed for three different nematode strains, across two different dose-rate treatments (Fig 2). This finding suggests that *P*. *hermaphrodita* might constitute a potential biodiversity threat to gastropod species endemic to the Pacific Northwest of North America. However, the effects of *P*. *hermaphrodita* on *M*. *fidelis* and other gastropods in laboratory infectivity assays might not accurately predict impacts in nature. Thus, additional studies examining nematode effects in other environments, such as in mesocosm settings, are required to further assess potential impacts of gastropod-killing nematodes on non-target hosts.

Survival rates for *M*. *fidelis* exposed to *P*. *hermaphrodita* (LT50 ranging from 20–42 days) were much higher than that observed for *D*. *reticulatum* exposed to *P*. *hermaphrodita* (LT50: 4–5 days) in essentially identical infectivity assays done in our labs [30]. The survival rates observed here for *M*. *fidelis* were also much higher than rates observed for *Testacella haliotidea* (an earthworm-eating subterranean slug species, native to Europe and introduced in the United States) exposed to *P*. *hermaphrodita* in similar assays done by our labs [31]. It is possible that the *M*. *fidelis* shells might play a protective role against parasitic nematodes as has been observed in other snail species [15], though further experimentation is required to assess this possibility. The broader factors underlying differences in *P*. *hermaphrodita*-induced mortality rates among different gastropod hosts require further analysis. We also observed variation among [strain x dose-rate] treatments within the present study (Fig 2B), though there was no clear pattern underlying this variation.

Infected snails with larger-diameter shells experienced significantly higher survival rates as compared to those with smaller-diameter shells (Fig 3), suggesting that smaller and less developmentally advanced *M*. *fidelis* are more susceptible to nematode-induced death as compared to hosts that are larger and more developmentally advanced. This pattern is consistent with observations in other snail species [15]. In tests of *P*. *hermaphrodita* on *Theba pisana*, *Cernuella virgata*, *Cochlicella acuta* and *Cochlicella* (= *Prietocella*) *barbara*, Coupland 1995 also found that smaller snails died more quickly than larger snails [32]. Other species that are susceptible at the juvenile stage include the slugs *Arion ater* [33] and *Arion lusitanicus* [34, 35] and the snail *Cornu aspersum* (= *Helix aspersa*) [21]. Based on our observations and previous research, we advocate for the inclusion of multiple host sizes and/or life stages, especially juvenile stages, in experimental infectivity assays in order to gain complete and unbiased insights into the susceptibilities of hosts to test biocontrol nematodes.

Despite limited knowledge on non-target effects of *P*. *hermaphrodita* and other nematodes in this genus, as recently pointed out by Christensen et al. 2021 [2], a narrative persists in the scientific literature that these nematodes pose minimal risk to non-target species [19, 36–39] (Bathon 1996; Wilson et al. 2000; Morand et al. 2004; MacMillan et al. 2009; Askary 2010). The findings described here adds to previous studies (Grewal et al. 2003; Rae et al. 2007) suggesting the potential for a wide host range for *P*. *hermaphrodita*, which contradicts assertions that this nematode species is of minimal concern to non-target slug and snail biodiversity. We advocate for caution in regulatory decisions surrounding the potential expansion of *P*. *hermaphrodita* and other biocontrol nematode species to new countries and their ecosystems until more is known about impacts on non-target hosts in their natural ecologies, especially for species native to world regions outside of Europe. We also encourage the expansion of biocontrol nematode non-target testing to include many phylogenetically diverse hosts, at many different life stages.

## Supporting information

S1 File (XLSX)
